# Psychotherapists’ Experience with In-Session Use of Routine Outcome Monitoring: A Qualitative Meta-analysis

**DOI:** 10.1007/s10488-024-01348-4

**Published:** 2024-03-20

**Authors:** Klára Jonášová, Michal Čevelíček, Petr Doležal, Tomáš Řiháček

**Affiliations:** Department of Psychology, Faculty of Social Studies, Masaryk University, Joštova 10, 602 00 Brno, Czech Republic

**Keywords:** Routine outcome monitoring, Qualitative meta-analysis, Therapists’ experience, Clinicians’ perspective, In-session use

## Abstract

Routine outcome monitoring (ROM) has become an increasingly utilized tool in therapeutic practice that has the potential to improve therapy outcomes. This study aimed to synthesize the findings of existing qualitative studies investigating how clinicians use ROM in their work with clients. A systematic search of qualitative studies on clinicians’ experience with the use of ROM in mental health services was conducted via PsycInfo, PsycArticles, Medline, Web of Science, and Scopus databases. Qualitative meta-analysis was used to synthesize the finding of the primary studies. Forty-seven studies met the inclusion criteria. The analysis resulted in 21 meta-categories organized into six clusters, namely (1) obtaining clinically relevant information, (2) adapting treatment, (3) facilitating communication, (4) enhancing the therapeutic relationship, (5) facilitating change in clients, and (6) personalized usage of ROM. The meta-analysis revealed that clinicians utilized ROM in diverse ways, including both informational and communicational functions. From the clinicians’ perspective, ROM was an element that, on the one hand, introduced additional structure and standardization in treatment and, on the other hand, allowed for greater flexibility and tailoring of treatment.

**Supplementary Information:**

The online version contains supplementary material available at 10.1007/s10488-024-01348-4.

## Introduction

Routine outcome monitoring (ROM) relies on the use of client-reported standardized measures at regular intervals to monitor patients’ progress in psychotherapy. The information obtained provides feedback to clinicians on client improvement and on processes that contribute to improvement (De Jong & Aafjes‐van Doorn, 2022). Several other terms have been used for this practice, including progress monitoring, clinical feedback, patient-focused research, feedback‐informed treatment, and measurement‐based care (Aafjes‐van Doorn & De Jong, 2022; Castonguay et al., 2013). ROM takes various forms ranging from simple paper-and-pencil methods to sophisticated software designed to provide therapists with statistically processed data on clients’ progress (De Jong & Aafjes‐van Doorn, 2022; Lutz et al., 2022).

In their systematic review, Lyon et al. (2016) identified 49 measurement feedback systems used in behavioral health care settings that differ in their characteristics and capabilities. While most of them track standardized outcomes, only a few allow clinicians to track idiographic measures and therapeutic processes. They also differ in terms of the feedback they provide (e.g., immediacy of feedback, comparison to normative data, generating predictions, and providing alerts). In the field of psychotherapy, the most widely known ROM systems include OQ Analyst, Partners for Change Outcome Management System and its derivatives, CORE Net, and Outcome Referrals. They differ both in the core outcome measure they use and in the inclusion of process variables, such as the working alliance.

The primary aim of ROM is the improvement of psychotherapy outcomes, and empirical evidence suggests that it has such potential (Rognstad et al. (2023). In their meta-analysis of 58 studies, De Jong et al. (2021) found that immediate systematic feedback on treatment progress increased the effectiveness of therapy and reduced drop-out. ROM was found to be especially useful in preventing negative treatment outcomes by reducing the number of deteriorating cases (Lambert et al., 2018; Shimokawa et al., 2010), probably because a stark difference between the expected course of treatment and the client’s progress provides more valuable information compared to patients who progress according to expectations (Kendrick et al., 2016).

The effect of ROM seems to depend on many factors. For instance, Bickman et al. (2011) found that ROM is more effective if it is provided at each session rather than every 90 days. De Jong et al. (2021) suggested that since the most significant change occurs at the beginning of treatment, it may be advantageous to use ROM more intensively in the initial phase of treatment and reduce the frequency later. Clinicians also tend to respond faster to feedback if they receive an alert from the ROM system (Douglas et al., 2015). Furthermore, ROM systems seem to work better when they are used directly in session and feedback is provided to both the client and the therapist (De Jong et al., 2012; Krägeloh et al., 2015). Barkham et al.’s (2023) review concluded that clients tend to support ROM when it is well-integrated in the treatment and when they understand its purpose. Rather, it is organizational, personnel, and resource issues that represent the greatest obstacles to the successful implementation of ROM. Despite its positive effects, the use of ROM in clinical practice remains an exception (Ionita & Fitzpatrick, 2014) and one of the essential variables determining ROM use is the clinician and their attitude towards ROM (Jensen-Doss et al., 2018).ROM may be effective via multiple mechanisms. On the informational level, ROM helps generate valid case formulations and formulate hypotheses about specific change mechanisms by providing clinicians with nuanced and systematic information on case development (Carlier et al., 2012). In this way, ROM can not only help clinicians correct their biases and “blind spots” (Janse et al., 2023; Macdonald & Mellor-Clark, 2015) but also provide useful information to clients themselves (Poston & Hanson, 2010). On the relational level, sharing feedback can strengthen the collaborative nature of the therapeutic relationship (Carlier et al., 2012). Brattland et al. (2019) found that the effect of ROM on treatment outcomes was mediated by an increase in the working alliance. Thus, the effect of ROM seems to be multifaceted, and a thorough understanding of how clinicians work with ROM in their practice may elucidate even more aspects of this complexity.

Qualitative studies on clinicians’ experience with using ROM have a great potential to provide detailed knowledge on the processes and experiences employed in using ROM, and thus complement the quantitative information on ROM effectiveness. While each qualitative study provides an in-depth probe into a small sample’s experience, a qualitative meta-analysis (QMA) allows researchers to synthesize the findings and provide a concise and comprehensive overview of the results, which can enable new insights or conceptualization of the researched topic (Timulak, 2009).

To date, two systematic reviews on the topic were published. Boyce et al. (2014) conducted a systematic review of qualitative research on professionals’ experience with ROM. Based on 16 studies, they identified four main themes, including the practical (collecting and incorporating data), attitudinal (valuing data), methodological (making sense of data), and impact (using data to make changes) aspects of using ROM. However, their study focused broadly on health care professionals, and some nuanced aspects relevant to psychotherapy and counseling may have been overlooked. Furthermore, their study is outdated because it does not cover the last decade, which has involved prolific research on ROM. Låver et al. (2023) analyzed 31 qualitative studies that explored clinicians’ and/or clients’ experience of using patients’ self-reported data in psychotherapy. They reported four main categories including (1) nomothetic uses (i.e., obtaining objective markers for assessment, process monitoring, and treatment planning); (2) intrapersonal uses (i.e., enhancing self-awareness, initiating reflection, and influencing patients’ mood or responses); (3) uses that prompt interactional processes (i.e., facilitating communication, supporting exploration, creating ownership in patients, changing treatment focus, enhancing therapeutic alliance, or disturbing the psychotherapy process); and (4) patients responding for specific purposes due to uncertainty and interpersonal motives or strategic responding to achieve a desired result. While Låver et al.’s study represents an up-to-date and more comprehensive synthesis, it did not specifically target the clinicians’ perspective, but rather combined the clinicians’ and clients’ perspectives into a more general, objectivist account. While this approach has merit on its own, it did not allow the authors to systematically distinguish between therapists’ clinical intentions and client-reported impacts.

### Aims of the Study

Monitoring clients’ progress and using this information as instantaneous feedback within treatment has the potential to improve treatment outcomes. However, the effect of ROM most likely depends on how clinicians implement ROM in their practice. Since clinicians serve as “gatekeepers” in this process, learning from their experience may elucidate the conditions and mechanisms of ROM effects. Using the method of qualitative meta-analysis, this study aimed to synthesize the findings of existing qualitative studies investigating how clinicians use ROM in their work with clients. Unlike Låver et al. (2023), our study focused specifically on the *clinicians’* perspective, providing a more detailed account of how clinicians reflect on the use of ROM. The study was preregistered with the PROSPERO database (anonymized).

A preliminary analysis of the data revealed that the findings can be divided into two thematic domains: (1) how clinicians integrate ROM in their in-session work with clients and (2) what kind of facilitators and barriers clinicians experience during ROM implementation. Due to the breadth of the findings, we focused on the former area and retained the latter for a subsequent study.

## Method

We used the Enhancing Transparency in Reporting the Synthesis of Qualitative Research (ENTREQ; Tong et al., 2012) guide to structure the report of the study. See Supplement 1 for the checklist.

### Selection of Studies

To be included in our meta-analysis, a study had to qualitatively examine therapists’ experience of using ROM in therapy. The study selection process followed the guidelines recommended by Timulak (2013; Timulak & Creaner, 2023). The PsycInfo, PsycArticles, Medline, Web of Science, and Scopus databases were searched for primary studies using the following search string: TI (monitoring OR feedback OR routine outcome) AND AB (qualitative OR thematic OR phenomenological OR narrative OR mixed method OR interview) AND AB (*therapist OR clinician OR counsellor OR counselor OR practitioner). The suitability of these terms was determined based on a study of the relevant literature. The search was conducted on November 15, 2022. Additional studies were identified by examining the references of all eligible primary studies.

Studies were included if they were (1) qualitative or mixed-method (in the latter case, only the qualitative parts were included), (2) based on the perspective of psychotherapists (including trainees) as users of routine outcome monitoring systems, (3) conducted in the context of psychotherapy or counseling, and (4) based on a formalized routine outcome monitoring/feedback system. All client populations (i.e., children, adolescents, and adults) and treatment modalities (i.e., individual, couple, group, and family therapy) were included.

A.A. conducted the screening. First, she removed duplicates and assessed the remaining studies based on the relevance of their titles. Second, she reviewed the abstracts of the remaining studies. Third, she read the full text of studies not excluded in the first two steps and assessed their suitability based on the inclusion criteria. The steps were than independently repeated by B.B. and discrepancies were discussed until consensus was reached.

The methodological quality of the primary studies was assessed based on Harden et al.’s (2004) criteria. These included the presence of (1) an explicit theoretical framework and/or literature review, (2) aims and objectives, (3) a clear description of context, (4) a clear description of the sample and how it was recruited, (5) a clear description of methods used to collect and analyze data, (6) attempts made to establish the reliability or validity of data analysis, and (7) inclusion of sufficient original data to mediate between evidence and interpretation. Each criterion was scores as 1 (present) or 0 (absent). A study could thus receive a total score between 7 (all criteria fulfilled) and 0 (no criterion fulfilled). The assessment was conducted by A.A. and reviewed by D.D.

### Data Preparation

A.A. extracted data on methodological aspects of the primary studies, including the study’s focus, clients’ presented issues, number, age, and ethnicity of clinicians, clinicians’ theoretical orientation, professional experience in general and with ROM in particular, the study’s context, the name of the ROM used, and the methods of data collection and analysis.

Once the studies were selected, A.A. thoroughly examined the Results sections of the original studies and extracted all findings related to clinicians’ experience with ROM within treatment that were presented as categories, descriptions, or participants’ quotes. If the study also reported clients’ experiences, only those reported by clinicians were included. A.A. also examined the Discussion sections of the primary studies. If they contained material that would typically be reported in the Results section (i.e., raw findings and participants’ quotes), she extracted them as well. All relevant findings were gathered into a single document for the meta-analysis.

### Data Analysis

First, A.A. divided the document with the extracted findings from the primary studies into meaning units, i.e., into phrases, sentences, or paragraphs with a coherent meaning (Rennie et al., 1988) which facilitates the coding process and coders’ discussions. Second, to develop an initial list of meta-categories, A.A. randomly selected five studies and inductively generated meta-categories by observing similarities and differences among the meaning units. Third, C.C. and D.D. revised this tentative categorization, and then the entire authors’ team discussed all ambiguities and suggestions for alternative coding to reach consensus about the emerging meta-categories and their definition. Within their discussion, they capitalized on the heterogeneity of their backgrounds and strived to develop a perspective that would make sense to all the co-authors. Fourth, to further develop and refine the list of categories, A.A. analyzed another 15 studies and, again, discussed the categorization with C.C. and D.D. to find consensus. Fifth, A.A. applied the meta-categories to the entire dataset; this process was largely deductive, with minor refinements of the meta-category system and definitions. Finally, B.B. and T.R thoroughly audited the completed analysis to search for potential inconsistencies and the whole team then settled on the final categorization. As already mentioned in the Aims of the Study section, this study focused on the first of the two thematic domains identified in the data (i.e., how clinicians integrate ROM in their in-session work with clients) and the second domain (i.e., what kind of facilitators and barriers clinicians experience during ROM implementation) is to be reported in a subsequent study. This decision was made during the analysis, after we developed a clear idea of primary studies’ content (Step 4). All primary studies described in this study pertain to this thematic domain.

### Reflection on Analysts’ Background

A.A. is a Ph.D. candidate at Masaryk University, Czech Republic, focusing on qualitative psychotherapy research. She is largely influenced by the humanistic and integrative traditions in psychotherapy. B.B. is a psychologist with 10 years of experience focusing mainly on psychotherapy research and qualitative methodology in an academic setting. His view of psychotherapy has been mostly influenced by integrative approaches. C.C. is a psychologist and psychotherapist with 10 years of therapeutic experience and has experience in qualitative research. He was trained in the systemic/postmodern tradition but has been influenced by an evidence-based approach to psychotherapy. He trains therapists in using ROM. D.D. is a psychologist and psychotherapist with 15 years of part-time therapeutic experience and an interest in both qualitative and quantitative research. He was initially trained in Gestalt therapy and was considerably influenced by the integrative movement. The last three authors were involved in the development of a ROM system.

## Results

**Fig. 1:**
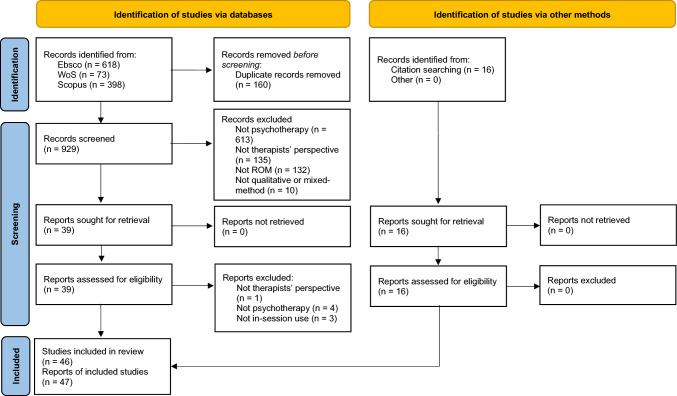
Flow diagram of the data retrieval

Our search strategy yielded 1089 reports, 47 of which met the inclusion criteria (see Fig. 1 for the flow diagram). Two reports were based on the same study.

### Description of the Primary Studies

See Supplement 2 for the list and detailed characteristics of the primary studies included in this meta-analysis. The studies were published between 2001 and 2023 and included a total of 1974 clinicians. The sample sizes varied between 1 and 324, with a median of 18. The samples were based in the US (n = 13), UK (n = 9), Australia (n = 5), Norway (n = 5), Netherlands (n = 3), Portugal (n = 2), Canada (n = 1), Chile (n = 1), Denmark (n = 1), and New Zealand (n = 1). One study included an international sample, and five studies did not report this information.

The populations treated included adults (n = 14), children and adolescents (n = 10), and mixed populations (n = 8). In 15 studies, this information was not reported. The treatments were provided in a variety of settings, including outpatient and inpatient facilities, and multiple facilities’ clinics were often included in a study.

The clinicians’ levels of experience in general and with ROM in particular, were reported inconsistently across studies, which did not allow us to summarize this information. Theoretical orientations, when reported, typically included a mixture of orientations. The primary studies seldom reported the names of the ROM system used. Instead, they presented a list of measures used in the study. Generally, very few details were provided in terms of what the ROM setup and feedback looked like.

In 70% of the primary studies, data were collected via individual interviews. In the remaining studies, data collection methods included focus groups, group discussions, open-ended surveys, written responses, and their combinations. In terms of data analysis, the studies used thematic analysis (n = 17), unspecified qualitative analysis (n = 9), content analysis (n = 8), grounded theory (n = 5), consensual qualitative research (n = 2), inductive constant comparison method (n = 1), narrative analysis (n = 1), phenomenological analysis (n = 1), and matrix analysis (n = 1).

Overall, the methodological quality of the primary studies was acceptable. On a seven-point scale, 39 of the 45 studies reached a score of 5 or higher. The most problematic aspect was a clear description of the sample and its recruitment (only satisfactory in 11 studies). See Supplement 3 for the methodological quality assessment.

The resulting meta-categories represent specific therapeutic intentions (or purposes) connected with the use of ROM. Although the meta-categories’ names may imply a positive effect (e.g., Opening and Speeding Up Discussion), the data cannot “prove” such an effect in any objective sense. Rather, the meta-categories capture *clinicians’ perspectives* on how they use ROM and how they reflect upon the role of ROM in the treatment. In fact, some clinicians expressed explicit concerns about negative effect in the case of several meta-categories. For instance, while ROM was used as a “structure reminder” to assist a therapist in keeping the treatment’s focus, some clinicians believed that the very same activity could distract a client from a personally meaningful topic. We included these concerns within the respective meta-categories. The meta-categories are summarized in Table 1.

**Table 1:** Summary of meta-categories

Meta-category	Description	Illustrative quote	n	%
*Cluster 1: Obtaining clinically relevant information*				
Assessing clients’ momentary status	ROM has become a part of the assessment process, allowing clinicians to assess the severity of clients’ problems, evaluate the risk of harm and/or self-harm, obtain hints about areas that require further exploration, estimate the likelihood of relapse, generate clinical hypotheses, and make diagnoses	“Many therapists thought of it as a type of radar that detected several issues that otherwise would stay hidden; for example, issues that patients found difficult to address in a session, and issues that therapists would never think of exploring.” (Hovland et al., 2023, p. 13)	30	64
Assessing clients’ progress	ROM was used to detect patterns of change, including improvement, stagnation, and deterioration	“[T]he advantage I see is the issue of, from there, also quantifying evolution, of perceiving how things are going in a more visible way and in a way and more, hum, concrete, isn't it?” (Dias et al., 2016, p. 6)	23	49
*Cluster 2: Adapting treatment*				
Treatment planning	ROM was used as a tool for treatment planning (e.g., to select a suitable treatment approach)	“I tend to use that (BASIS-32) when talking about the individual service plan and … you get them to run through that and then you will look at that and say—well OK you feel that this one really is a big thing that we need to work on collaboratively.” (Callaly et al., 2006, p. 168)	18	38
Adjusting the treatment process	Ongoing feedback about treatment impact allowed clinicians to adjust the treatment course whenever needed (e.g., intensify or terminate the treatment)	“I have had many sessions change because of a discussion based on an answer they had to on [the MFS], and I don’t think I would have found that information if we had just done a normal session.” (Sichel & Connors, 2022, p. 11)	16	34
Structure reminder	The regularity of ROM helped clinicians maintain a treatment structure. Apart from the routine nature of the measurement itself, ROM contributed to keeping the treatment goal-oriented and served as a reminder of important topics	“ROM focuses the mind of the practitioner…look at goals and if they’ve been met.” (Norman et al., 2014, p. 584)	7	15
Case management	ROM facilitated case management, for instance, by prompting clinicians to supervise a client or providing information staff meetings	“We pull up the graph, go into the client's record and see what their progress has been like. And from then my supervisor will ask me if there have been any potential barriers, anything that got in the way of the client progressing on track as we would hope to expect, and it helps me to be able to reflect.” (Delgadillo et al., 2017, p. 15)	9	19
*Cluster 3: Facilitating communication*				
Opening and speeding up discussion	ROM tools made it easier for clinicians to open conversations about clients’ concerns and perspectives on the therapeutic work	“This [GQ intervention] seemed to enable the group members to jump right into a discussion of the previous session and what they wanted from each other.” (Whitcomb et al., 2018, p. 138)	27	57
Supporting difficult conversations	ROM helped clinicians normalize and support conversations on difficult topics, such as client deterioration, the therapeutic relationship, and various potentially stigmatizing themes (e.g., self-harm)	“I think the form gives permission for them to express things that they maybe are not quite so satisfied with, you know … It’s like you are explicitly saying to them, ‘you know, it’s ok to say this’, and the fact that they are not having to raise it themselves—you’re initiating and giving them a structure—I think is helpful.” (Bowens & Cooper, 2012, p. 57)	8	17
*Cluster 4: Enhancing the therapeutic relationship*				
Allowing clients to feel heard	Clinicians used ROM to invite clients to voice their wishes, thoughts, and reactions	“[O]ne of the therapists explained that his patient felt ‘better cared for’ and ‘more contained’ thanks to the experience of completing the measures every session.” (Errázuriz & Zilcha-Mano, 2018, p. 135)	8	17
Enhancing transparency in the therapeutic relationship	ROM helped clinicians promote transparency in the therapeutic relationship by facilitating conversations on the therapeutic relationship	“For me the biggest thing is about the increase in the collaboration with yourself and the patient, also creating a transparency of what these measures are for, I found that helpful, and it boosts the relationship.” (Delgadillo et al., 2017, p. 16)	13	28
Facilitating clients’ involvement	ROM became a means of enhancing clients’ autonomy, participation in treatment-related choices, and responsibility for the treatment	“I would show [the report] to them, and I would try to explain it and everything. ‘And this is what the data says…’ in terms of engaging them into their treatment, it’ll help with that…this is…shared decision making. And this gives them more of a connection and participation in their treatment.” (Stefancic et al., 2022, p. 10)	10	21
Nonspecific impacts on the therapeutic relationship	This meta-category contains general statements about the perceived, mostly positive, influence of ROM on the therapeutic relationship	“Actually, [the feedback] has got me thinking about one aspect. I used to be heavily worried about technique and if I was using the right techniques and interventions, and now I’m focusing more on the relationship aspect.” (Dayton, 2011, p. 75)	24	51
*Cluster 5: Facilitating change in clients*				
Facilitating insight in clients	Clinicians used ROM to facilitate insight in clients, for instance, about the course of their illness	“It’s good—using those graphs and taking them back to the client you can show them that they have improved—I think that for this particular reason they are useful over a longer period of time especially.” (Callaly et al., 2006, p. 168)	12	26
Keeping clients focused	ROM helped clinicians remind clients of their goals and keep a focus on these	“Both patients and therapists were unanimously enthusiastic about the suggestion for monitoring personal treatment goals. Patients thought that it would help them to focus on their goals, it would motivate them to reach their goals and be easy to understand. Therapists responded in a similar way.” (Koementas-de Vos et al., 2022, p. 211)	3	6
Reinforcing positive change	Clinicians believed that ROM output could reinforce positive change in clients by reaffirming that therapy helps	“I love being able to give them numbers and say ‘this is how much better you’ve gotten.’ It’s kind of awesome. Like yesterday I had a client discharging and I was able to talk to him about it, like ‘do you see how much work you’ve really done?’ …Use it for empowering.” (Brooks Holliday et al., 2021, p. 216)	7	15
Other therapeutic impacts	This meta-category contains other, marginally mentioned impacts or purposes of use, such as using ROM as a means of externalization	“The separation of problems and persons is embedded in this way of talking. …One literally placed the development on the table in the form of scores and curves, and through this distance was created. One could then relate the questions and conversation toward what the scores and curves represented. As visualizations, these became a focus of joint attention for the participants: ‘…you can talk about it as something down here (knocks on the table), not as something inside you’.” (Sundet, 2012, p. 127)	2	4
*Cluster 6: Personalized usage of ROM*				
Adapting timing of administration	Clinicians differed in the frequency and regularity with which they used ROM with their clients, as well as in how they integrated the measurement within the session	“We initially started doing that every session but because some of the clients are here for a long time, that got to be cumbersome and we also found we were collecting more data than we were using.” (Ionita et al., 2016, p. 176)	15	32
Adapting mode of administration	Clinicians reported on how the adapted the mode of administration to their own, as well as clients’ needs and preferences	“Sometimes people have reading difficulties or they’re not confident using the computer’ and the therapist may sit side by side …just clicking all the screens right in front of the client and reading out the questions and clicking for them.” (Unsworth et al., 2012, p. 76)	10	21
Adapting how feedback is discussed with clients	Clinicians varied in the extent to which they discussed feedback with clients	“There was no established or uniform way of managing the feedback report. Some clinicians discussed the report with their patients, some just read the report, and others considered how to use the report from session to session.” (Hovland et al., 2023, p. 12)	5	11
Focusing on specific aspects	Some clinicians stated that they did not use ROM output in its entirety, but selectively focused on some aspects of the report	“I sometimes score it before I start a session, to review with the patient because I am concerned, but most of the time the scores are right in the middle or toward the none or very seldom. So those I just skim through, or item number 9, which is about suicidal thoughts and then I pay attention to that when I ask ‘what is this about,’ regardless of whether I score [the measure] or not … I may use this as a reference, but I do not spend too much time on them.” (Brooks Holliday et al., 2021, p. 216)	8	17
Contextual interpretation	Clinicians tended to interpret ROM results in the context of other information they had about the case, such as clients’ life circumstances	“If you try to use a lot of standardized things then you may miss things that they don’t measure. You are only measuring the things that are included in the diagnostic assessment. That means that you are forced to look at things from a medical view point.” (Martin et al., 2011, p. 416)	12	26

N(%) = the number (percentage) of studies within which the meta-category was identified. These frequencies reflect the salience of the categories across primary studies. They by no means represent the prevalence of the types of experience across psychotherapists because qualitative studies are not designed to represent the prevalence of categories in the population. For a more detailed report of which meta-category was found in which study, see Supplement 4

### Cluster 1: Obtaining Clinically Relevant Information

#### Assessing Clients’ Momentary Status

For some clinicians, ROM has become a part of the assessment process, allowing them to assess the severity of clients’ problems, evaluate the risk of harm and/or self-harm, obtain hints about areas that require further exploration, estimate the likelihood of relapse, generate clinical hypotheses, and make diagnoses. ROM served this purpose not only as part of the initial assessment but also during the whole course of the treatment: “even before they come here, you have an idea of, eh, is the person coming here and have felt really, really bad since last time, or does it look reasonable?” (Tarp et al., 2022, p. 7). Clinicians especially appreciated discovering information that would normally be overlooked in the dialog with the client and considered ROM “a type of radar that detected several issues that otherwise would stay hidden; for example, issues that patients found difficult to address in a session, and issues that therapists would never think of exploring” (Hovland et al., 2023, p. 13). They were sometimes able to identify the incongruences between clients’ experiences and ROM outcomes, which then became potential sources of new insights. Ultimately, it was the clinicians who decided whether and how to use the information obtained from ROM based on their expertise and clinical judgment.

#### Assessing Clients’ Progress

Measuring outcomes across time allowed clinicians to detect various patterns of change, including improvement, stagnation, and deterioration. Clinicians appreciated that thanks to ROM, information on treatment progress was “systematic and accurate” (James et al., 2015, p. 6), “measurable and specific” (Koementas-de Vos et al., 2022, p. 208), and “visible” and “concrete” (Dias et al., 2016, p. 5). This gave them an opportunity to have “a view of what is going on” and see “the impact of what [they] are doing” (Norman et al., 2014, p. 581). In cases of no progress, clinicians used the data to explore potential causes with the client. However, some clinicians also worried that “in psychotherapy, the changes are not so evident, so straightforward or immediate” (Norman et al., 2014, p. 589). They argued that immediate subtle changes in the long-term treatment impact are difficult to measure.

### Cluster 2: Adapting Treatment

#### Treatment Planning

Clinicians reported using ROM as a tool for treatment planning. They used the scores to get “ideas before approaching the case” (Martin et al., 2011, p. 413) regarding the type of difficulties clients had and to get “a sense of what kind of therapy someone is looking for” (Bowens & Cooper, 2012, p. 55). This helped them “select a certain therapeutic approach [such as] behavioral activation vs. cognitive therapy” (Brooks Holliday et al., 2021, p. 218) for a given client. In some institutions, ROM became a part of the formal procedure of individual service planning. Although ROM outputs were used to inform treatment direction, clinicians nonetheless tended to believe that “the ultimate autonomy and flexibility of use should lie with the clinicians themselves” (Sharples et al., 2017, p. 223).

#### Adjusting the Treatment Process

Ongoing feedback about treatment impact allowed clinicians to adjust the treatment course whenever needed. For instance, clinicians were able to detect harmful and unhelpful processes, intensify the treatment, make a decision about treatment termination, and better tailor the treatment to clients’ needs. Some of them believed that this would not be possible without ROM: “I don’t think I would have found that information if we had just done a normal session” (Sichel & Connors, 2022, p. 11). In addition to merely providing information that guided the adjustment, many clinicians reflected on “*a sense of permission or freedom* to alter their practice in particular ways as a result of (…) clients’ feedback” (Bowens & Cooper, 2012, p. 56, emphasis added).

#### Structure Reminder

ROM provided structure for the treatment in several ways. First, regular measurements created routine in the therapeutic process. Second, ROM helped make treatment goal-oriented by allowing clinicians to “keep a focus on whether treatments/interventions are meeting these goals” (Norman et al., 2014, p. 583). Third, ROM served as a reminder of “important topics to deal with and made starting a relevant conversation easier” (Hovland et al., 2023, p. 12). Some clinicians reported using ROM data to “prepare what was going to be the focus of the forthcoming session” (Tarp et al., 2022, p. 7). However, some clinicians also worried that ROM could become a distractor that may deflect “attention and focus away from the central agenda of the therapy toward aspects of the scales” (Sundet, 2012, p. 125).

#### Case Management

Clinicians perceived ROM as a tool that facilitated case management. ROM output served both as a prompt to supervise a client and an input for the supervision. Furthermore, it was instrumental in staff meetings and interdisciplinary case review meetings: “During staff meetings, the ROM results are presented, and when they demonstrate lack of progress, different courses of action are discussed” (De Beurs et al., 2011, p. 7). ROM data were also used to justify a referral.

### Cluster 3: Facilitating Communication

#### Opening and Speeding Up Discussion

ROM tools made it easier for clinicians to open conversations about clients’ concerns and their perspectives on the therapeutic work. On the one hand, the standardized format of ROM tools facilitated responding for some clients, as it was “easier for some to tick a box or give a score out of ten than to explain it to [the clinician] in words” (Martin et al., 2011, p. 414). On the other hand, the tools served as a springboard for a dialog that could lead in different and sometimes unexpected directions. As one clinician noted, ROM “can sometimes open the door for me to talk about something… sometimes it does give me an answer to something that I would have otherwise found out too late” (Garland et al., 2003, p. 400). In this way, ROM was able to speed up the therapeutic process and save time, especially during the initial interview.

Once clinicians had clients’ responses on ROM items, it was easier for them to pursue further details. For instance, they asked clients to “elaborate on specific symptoms they endorsed on a given measure” (Brooks Holliday et al., 2020, p. 277) or negotiated “what patients intended to communicate” (Hovland et al., 2023, p. 13). Used in this way, clients’ responses to ROM items “became prompts to stimulate further discussion rather than definitive and objective indicators of progress” (Savic & Fomiatti, 2016, p. 182).

#### Supporting Difficult Conversations

ROM helped clinicians normalize and support conversations on difficult topics, such as client deterioration, the therapeutic relationship, and various potentially stigmatizing themes (e.g., self-harm): “[clinicians] shared the experience that a needed contribution from a feedback system was to allow for conversations about this important issue [trust], which is often avoided, postponed, or forgotten in the treatment process” (Moltu et al., 2018, p. 256). Some clinicians used ROM to encourage clients to express their dissatisfaction: “I think the form gives permission for them to express things that they maybe are not quite so satisfied with… It’s like you are explicitly saying to them, ‘you know, it’s ok to say this’” (Bowens & Cooper, 2012, p. 57).

### Cluster 4: Enhancing the Therapeutic Relationship

#### Allowing Clients to Feel Heard

Clinicians reported that ROM, by its very nature, invited clients to voice their wishes, thoughts, and reactions. In this way, clinicians could hear clients’ priorities and try to align with their perspective. They appreciated this as a means of becoming more empathetic and believed that their clients “felt ‘better cared for’ and ‘more contained’ thanks to the experience of completing the measures every session” (Errázuriz & Zilcha-Mano, 2018, p. 135). As one clinician reported, “asking and understanding what the clients need in session has aided me in creating a stronger alliance with clients because … it sends a message that we are here for them and want to help them as best as we can” (Esmiol-Wilson et al., 2017, p. 32).

#### Enhancing Transparency in the Therapeutic Relationship

For some clinicians, using ROM promoted the perceived transparency in the therapeutic relationship by encouraging “more open relating” (Bowens & Cooper, 2012, p. 58) and helping clients “feel safe enough to share their negative feedback” (Esmiol-Wilson et al., 2017, p. 28), thus “making direct interpersonal communication easier” (Moltu et al., 2018, p. 256). Not only did it help clinicians invite clients’ voices and explore moments in which “they were off-point, misunderstanding, or insensitive to important aspects of the patients’ experiences” (Moltu et al., 2018, p. 256), ROM also allowed them to be more transparent and authentic themselves. For instance, they felt less inhibited to show their “personality and humour” (Bowens & Cooper, 2012, p. 56) and to “be more challenging” (Bowens & Cooper, 2012, p. 56) when the client specifically asked for it in feedback.

#### Facilitating Clients’ Involvement

From the clinicians’ perspective, ROM became a means of enhancing clients’ autonomy, participation in treatment-related choices, and responsibility for the treatment. In this sense, ROM facilitated the collaborative aspect of the treatment, enhancing “an element of ‘mutuality, where you’re both working on the same goal’” (Bowens & Cooper, 2012, p. 58) and a sense of “shared decision-making” (Stefancic et al., 2022, p. 9). However, ROM did not have this effect on its own: “[ROM], by itself, cannot achieve a shift toward service‐user empowerment …[because] realizing such potential depends on participants’ active negotiations during sessions” (Hovland et al., 2023, p. 11). On a critical note, one study echoed a clinician’s concern that clients may become burdened by excessive responsibility placed on them by asking them about their treatment preferences: “it may be giving the clients too much responsibility, particularly in the early stages of therapy” (Bowens & Cooper, 2012, p. 57).

#### Nonspecific Impacts on the Therapeutic Relationship

Several studies reported general statements about the positive influence of ROM on the therapeutic relationship. According to some clinicians, ROM can speed up the development of the relationship: “it closens the relationship before you’re even really started” (Bowens & Cooper, 2012, p. 58). Consequently, clinicians valued aspects of the feedback system that pertained to the alliance, “especially since participants described the early alliance work as delicate and vulnerable” (Lavik et al., 2020, p. 9). Paying attention to alliance-related information allowed them to respond to clients who showed signs of a poor therapeutic relationship. Some clinicians reported negative experiences, sharing that “ROM was intrusive, violating the privacy of the therapy dyad” (De Beurs et al., 2011, p. 7). However, the lack of contextual information did not allow us to determine whether this kind of report was a consequence of clinicians’ negative experience or if it resulted from their a priori pessimistic assumptions about ROM.

### Cluster 5: Facilitating Change in Clients

#### Facilitating Insight in Clients

ROM does not serve only as a source of information for clinicians. Using ROM data, clients and therapists can jointly “investigate the causal relations between symptoms and what might have triggered them” (Tarp et al., 2022, p. 7). Clinicians appreciated ROM output as a means of helping patients to “understand their course of illness and, thus, support psychoeducational aspects of the therapy” (Tarp et al., 2022, p. 7). Sharing the output with clients gave them “a sense of where they were[;] the graph reflected that” (Unsworth et al., 2012, p. 76). In the case of couple or family therapy, this increased the awareness of how other members of the family were doing: “following each other’s curves gave family members insight into the processes of change that other family members were going through” (Sundet, 2012, p. 126).

#### Keeping Clients Focused

For some clinicians, ROM became a tool that helped them remind clients of their goals: “monitoring helps patients to keep their goals actively in mind” (Koementas-de Vos et al., 2022, p. 211). ROM also helped clarify “which ones we have achieved, which ones are what we didn’t achieve and why” (Dias et al., 2016, p. 5).

#### Reinforcing Positive Change

Clinicians believed that ROM output could also serve to “motivate and reinforce positive change when patients’ symptoms were on track” (Delgadillo et al., 2017, p. 14). As Hovland et al. (2023) documented, “[J]ust looking at patients’ improvements on the feedback report could have a therapeutic effect, by giving hope or reaffirming that therapy helps” (p. 12).

#### Other Therapeutic Impacts

Other therapeutic impacts were mentioned only marginally; therefore, we did not develop specific categories for them. Sundet (2012) reported how ROM was used as a means of externalization: “One literally placed the development on the table in the form of scores and curves, and through this distance was created. One could then relate the questions and conversation toward what the scores and curves represented” (p. 127). One study also mentioned a potential negative impact of constantly reminding clients of their problems. To prevent relapse, clinicians sometimes decided to eschew using ROM: “clinicians often reproduced notions of client vulnerability to relapse in deciding not to administer the tool to clients who had left treatment: Sending them messages, ‘How many times have you had a joint in the last month?’ ‘I haven’t had any for 6 weeks and now you’ve just made me think about it” (Savic & Fomiatti, 2016, p. 178).

### Cluster 6: Personalized Usage of ROM

#### Adapting Timing of Administration

Clinicians differed in the frequency and regularity with which they used ROM with their clients. For instance, in Brooks Holliday et al.’s (2021) study, some clinicians “reported administering measures each time they saw the patient (whether weekly or less often) and the discussion in the treatment session was fairly brief” (p. 215), while others “reported administering measures less often but appeared to have a more thorough discussion regarding the meaning of scores” (p. 215). Furthermore, some clinicians used ROM “more often with patients during the initial phases of treatment, and transition to less frequent administration for their longer-term patients or those in maintenance phases” (Brooks Holliday et al., 2021, p. 276). Clinicians also varied in how they integrated the measurement within the session. Some preferred having the measurement done before the session so that it did not interfere with the therapeutic process, while others administered ROM during the session, either at a specific time within the session (e.g., beginning of the session) or “slotting ‘it where it flows’” (Unsworth et al., 2012, p. 76). Clinicians often emphasized that they adapted the timing of administration based on their clients’ needs and preferences. However, the use of ROM was also impacted by the fact that clinicians sometimes simply forgot to administer the measures.

#### Adapting Mode of Administration

Some clinicians mentioned choosing between an electronic and paper-and-pencil mode of administration, sometimes reflecting clients’ preferences. They also applied various individualized, and sometimes innovative, strategies to assist clients with completing ROM measures. These included reading the items out loud and entering clients’ responses in the system, mailing questionnaires to clients with a cover letter, and “working through several initial questions with the consumer and then allowing the consumer to complete the remainder” (Trauer et al., 2009, p. 149).

#### Adapting How Feedback is Discussed with Clients

Clinicians varied in the extent to which they discussed feedback with clients. While some “discussed each questionnaire item with patients at every session” (Lucock et al., 2015, p. 641), others “administered measures regularly but did not discuss the results frequently” (Brooks Holliday et al., 2020, p. 217), and yet others “just read the report” (Hovland et al., 2023, p. 12) without bringing it back to the client at all. Clinicians also differed in asking additional questions about the ROM results. Some preferred to “have a little conversation on what that was like, and what comes out of it” (Savic & Fomiatti, 2016, p. 182) or to ask questions about the supposed causes, such as, “Do you have an idea why your scores changed?” (Koementas-de Vos et al., 2022, p. 207), while others kept to the standard measures’ items. This category was present exclusively in studies on adult clients.

#### Focusing on Specific Aspects

Some clinicians stated that they did not use ROM output in its entirety, but selectively focused on some aspects of the report. They may have had specific items they regularly looked at (e.g., risk items, alliance, or social support), observed “changes on the graphs over time”, or paid attention to “whether something stood out” (Hovland et al., 2023, p. 12). Some clinicians expressed a wish to have certain types of items or instruments in their ROM or to have an opportunity to adapt the instruments to a client’s specific needs.

#### Contextual Interpretation

Clinicians tended to put ROM results in context. Some explicitly mentioned that “there is a danger in just focusing purely on outcome measures without any kind of interpretation and qualitative information” (Hall et al., 2014, p. 8). When relying on standardized measurements only, clinicians were afraid they would “miss things that they don’t measure” (Martin et al., 2011, p. 416). Therefore, they interpreted ROM outputs in the context of other information they had about the case. Life circumstances and case specificity could alter data interpretation significantly: “…when interpreted in the context of the client’s life circumstances, then this lack of change might be interpreted as progress” (Savic & Fomiatti, 2016, p. 176).

## Discussion

The purpose of this qualitative meta-analysis was to summarize how clinicians integrate ROM in their in-session work with clients. The results suggest that clinicians use ROM to enhance multiple treatment features, including clinical data collection, treatment planning, and important interpersonal processes, and that ROM can also serve as an intervention per se. The benefits of ROM appear to be influenced by the thoroughness with which clinicians make ROM an integral part of the treatment and by the flexibility of ROM tools matching the context of the case and clinicians’ skillset.

On a higher level of abstraction, clinicians used ROM for three purposes, namely informational, communicational, and structural. From the *informational* perspective, clinicians used ROM to obtain relevant data and to plan and adjust treatment. From this perspective, the core benefit of ROM was to provide clinicians with additional sources of clinical information and help them adapt the treatment course. This is in accordance with the research showing that ROM may help clinicians access the most important therapy themes and that ROM helps to reduce the chance that potential risks are overlooked (Lambert, 2010). This improved clinical reasoning is likely associated with the potential to reduce some natural cognitive limits that are pertinent to the profession (Macdonald & Mellor-Clark, 2015). Surprisingly, primary studies have rarely mentioned that ROM provides information on factors that contribute to treatment outcomes, such as change mechanisms, which would further help clinicians develop focused case formulations. The reason might be that most ROM systems predominantly focus on outcome monitoring because outcome measures are more available and refined for swift use in routine practice. Nevertheless, suitable process measures have been developed for this purpose and implemented in some ROM systems (e.g., Clinical Support Tools, Lutz et al., 2019, and Session Rating Scale, Duncan et al., 2003).

The *communicational* function of ROM includes stimulating the shared decision-making process in treatment, which can improve the working alliance (Youn et al., 2012); this is associated with improved treatment outcomes (Horvath & Bedi, 2002). However, if ROM should support shared decision-making and clients’ involvement in the treatment process, the clinician needs to create a culture of feedback within which the client is not afraid to express his or her concerns about the treatment process or the therapeutic relationship (Prescott, 2017). The potential of ROM to empower clients’ decision-making in treatment also depends on the therapist-client negotiation of the purpose of ROM during the session (Hovland et al., 2023). In addition to bolstering the alliance, our meta-analysis showed that clinicians recognized additional communicational ROM impacts in their in-session work, such as starting difficult conversations (e.g., about stigmatized issues, nonimprovement, and relational factors in treatment), opening and speeding up conversation about issues relevant for clients, and supporting transparent and attentive conversation. These impacts have been rarely mentioned as potential mediators of ROM effectiveness in research studies and they may deserve further attention.

Somewhat paradoxically, the *structural* purpose of ROM served clinicians to simultaneously maintain the treatment structure and exercise flexibility. From the perspective of *structure*, regular administration of measures became a ritual that structured therapeutic conversation. Furthermore, ROM served as a reminder of important themes and goals and helped clinicians and clients stay focused. Paying attention collaboratively to goals in therapy and monitoring progress toward these goals is associated with a better outcome (Tryon, 2018). This may be especially true when working with clients who prefer structured and goal-oriented approaches to psychotherapy (Cooper et al., 2019). However, ROM also stimulated clinicians’ *flexibility* by providing prompts to respond in a different way and changing the treatment direction in accordance with the clients’ current needs. This was often the result of obtaining information that might otherwise remain unnoticed by the clinicians. Tailoring treatment is assumed to reduce the risk of premature termination (Meier, 2015). Interestingly, some clinicians also noted that ROM became a kind of justification for them to be more flexible with their approach, allowing them to try additional interventions such as being more challenging or involved when clients expressed that they would welcome such an approach. However, embracing flexibility might not be easy for some clinicians, especially if it includes processing negative feedback (Boyce et al., 2014).

Clinicians’ flexibility in timing, frequency, and other administration factors that fit the treatment context parallels their ability to adapt the treatment itself when needed. Being allowed and able to adapt the data collection process (Boyce et al., 2014; Unsworth et al., 2012) and interpret the collected data contextually (Norcross & Wampold, 2019) seems to be essential for the effective use of ROM. Many clinicians noted that it was essential to “dose” administration and interpret the data with respect to the context of specific clients’ lives, like any other intervention. As noted by Prescott (2017), mastery of ROM knowledge and its use is not sufficient without the ability to integrate this knowledge into work with clients. Moreover, clinicians may tend to view outcome measures as less informative and reliable than their own clinical judgment (Jensen-Doss & Hawley, 2010); hence, they should be encouraged to integrate ROM output and their own clinical judgment. This presents at least two challenges. First, from the research perspective, it is necessary to examine how clinicians use ROM with specific types of clients in greater idiosyncratic detail, for instance, via case studies (De Jong & Aafjes-van Doorn, 2022). Second, from the perspective of practical application, the flexible use of ROM poses a challenge in finding an optimal in-session integration while maintaining its functionality with respect to, for example, the psychometric functioning of the measures.

There was little overlap between our findings and those of Boyce et al.’s (2014) meta-analysis. This was mainly because Boyce et al. focused on facilitators and barriers to successful ROM implementation while our study was focused on in-session use of ROM. The overlap of our findings with Låver et al.’s (2023) meta-analysis is more substantial. In their meta-categories, Låver et al. represented some aspects of almost all of our clusters. However, the narrower focus of our study allowed us to formulate several specific meta-categories not explicitly covered in Låver et al.’s study. These include *Structure Reminder* and *Case Management* (Cluster 2), *Allowing Clients to Feel Heard* and *Enhancing Transparency in the Therapeutic Relationship* (Cluster 4), and *Keeping Clients Focused*, *Reinforcing Positive Change*, and *Other Therapeutic Impacts* (Cluster 5). Furthermore, Låver et al. did not mention any aspect pertaining to the personalized usage of ROM itself, and therefore, the whole Cluster 6 is new. The findings of our study are also based on a larger body of primary studies (N = 47) than those of Låver et al.’s study (N = 31).

Both our and Låver et al.’s (2023) meta-analyses identified a diversity of purposes and potential impacts of ROM in the therapeutic process. As a response to this heterogeneity in ROM usage, several practice recommendations were developed, outlining factors linked to ROM effectiveness. For instance, Aafjes‐van Doorn and De Jong (2022) recommended (1) regular monitoring of relevant outcomes using repeated administration of measures; (2) using the data to inform treatment decisions; and (3) sharing collected data with clients and supervisors. Barber and Resnick (2022) developed similar recommendations that emphasized collecting, sharing, and acting on the collected data, with attention to shared decision-making with clients. In a broad sense, ROM fits within practice recommendations represented by the shared decision-making model in mental health care (Langer & Jensen-Doss, 2018) and the development of professional practice guidelines dedicated to ROM is needed (Boswell et al., 2023).

## Limitations

There was a large heterogeneity in the extent of the clinicians’ direct experience with ROM and this information was also inconsistently reported across the reviewed studies. As a result, it was difficult to distinguish reports of real impacts of ROM use from clinicians’ a priori assumptions (e.g., when clinicians talked about negative consequences of ROM usage for the therapeutic relationship, but they did not explicitly state whether they experienced such impacts in their practice). Therefore, the results may be influenced by clinicians’ expectations and reluctance, rather than their direct experience with systematic ROM use. To reduce this possibility, we strived to remove the excerpts that clearly represented mere assumptions (i.e., when clinicians stated what “might” happen, not what happened) in the analysis.

Our review might present a more agreeable view of ROM use in practice compared to the general community view, including the view of clients (Solstad et al., 2019). There are two reasons why the present results might sound more in favor of ROM. First, many clinicians were skeptical and voiced concerns about the negative effects of ROM in practice, often unrelated to direct experience with ROM. However, we reserved these perspectives for a subsequent study that will focus on facilitators and barriers (including clinicians’ a priori concerns) to ROM implementation. Another factor that contributed to a rather favorable view of ROM might be the potential allegiance of the primary studies’ authors to the ROM systems they investigated. Therefore, ROM implementation in practice might meet with more resistance from clinicians than the present study suggests.

The results of this review depend on the quality of the primary studies and the credibility of their findings. For some studies, there was a lack of detail in the methodological sections and the descriptions of the specific use of ROM within study results. There was also wide variability in the measures, ROM systems, and their implementation, which may limit clinicians’ ROM usage reflected in the results. While our findings capture the potential of ROM, they may not be directly transferable to every ROM system. However, heterogeneity can also be perceived as a strength since we were able to capture the variability in clinicians’ ROM in session integration.

The primary studies also differed in terms of how much they contributed to the analysis. The percentage of meta-categories to which each study contributed varied from 62 to 5% (*Mdn* = 24%). This means that no single study was able to capture the breadth of clinicians’ experience. However, studies that encompassed less categories were not automatically of lower quality (in fact, the correlation between the number of included meta-categories and the study’s methodological quality was negligible, *r* = 0.03). Furthermore, qualitative meta-analyses are considered resilient to the inclusion of an occasional weaker study (Levitt, 2018).

## Conclusions and Future Directions

The findings of the present meta-analysis highlight several themes relevant for the future development of ROM in clinical practice. First, ROM works through and due to clinician involvement. This notion may appear unsurprising because the impacts of any clinical tools and interventions depend on clinicians’ skillful integration in the treatment process. Nevertheless, this finding highlights the need to further explore the clinician variable and its role in the effective use of ROM (Miller et al., 2015; Wampold & Miller, 2023) because clinicians’ attitudes, skills, training, and institutional context determine whether ROM functions as an effective clinical tool. This appears even more important if we consider that ROM implementation may fail because of clinicians’ concerns about the negative impacts of ROM in their practice. Many such concerns appear to be reduced when ROM tools are offered in a flexible manner that allows clinicians to adapt them to their approach and to the context of their work. Second, clinicians who were motivated to integrate ROM in practice were able to use it as an intervention on its own. For instance, some used ROM to convey information about clients' progress, while others used ROM to empower clients. Exploring these intervention-like uses may further enrich the main purpose of ROM systems. Third, the monitoring of factors that contribute to the change process should become more available and on par with outcome measurement. This would help clinicians better understand “what” helps specific clients in addition to “how much” it helps. Moreover, the rich data collected in this way might further help us understand why, how, and whom therapy helps.

## Supplementary Information

Below is the link to the electronic supplementary material.

Supplementary file1 (DOCX 101 kb)
