# Supplementary material for: Psychotherapists’ Experience with In-Session Use of Routine Outcome Monitoring: A Qualitative Meta-analysis
Source: Adm Policy Ment Health. 2024 Mar 20;52(1):106–22. doi: 10.1007/s10488-024-01348-4 (PMC11703987; doi:10.1007/s10488-024-01348-4)
Supplement: Supplementary file 1 — Supplementary file1 (DOCX 101 kb) [file 10488_2024_1348_MOESM1_ESM.docx]

**SUPPLEMENTARY MATERIAL FOR**

**“Psychotherapists’ experience with in-session use of routine outcome monitoring: A qualitative meta-analysis”**

# Supplement 1. *The ENTREQ checklist (Tong et al., 2012)*

| No. Item | Guide questions/description | Reported on Page # |
| --- | --- | --- |
| 1. Aim | State the research question the synthesis addresses | 5 |
| 2. Synthesis methodology | Identify the synthesis methodology or theoretical framework which underpins the synthesis, and describe the rationale for choice of methodology (e.g.  meta-ethnography, thematic synthesis, critical interpretive synthesis, grounded theory synthesis, realist synthesis, meta-aggregation, meta-study, framework synthesis) | 7-8 |
| 3. Approach to searching | Indicate whether the search was pre-planned (comprehensive search strategies to seek all available studies) or iterative (to seek all available concepts until they theoretical saturation is achieved) | 6-7 |
| 4. Inclusion criteria | Specify the inclusion/exclusion criteria (e.g. in terms of population, language, year limits, type of publication, study type) | 6 |
| 5. Data sources | Describe the information sources used (e.g. electronic databases (MEDLINE, EMBASE, CINAHL, psycINFO), grey literature databases (digital thesis, policy reports), relevant organisational websites, experts, information specialists, generic web searches (Google Scholar) hand searching, reference lists) and when the searches conducted; provide the rationale for using the data sources | 6 |
| 6. Electronic Search strategy | Describe the literature search (e.g. provide electronic search strategies with population terms, clinical or health topic terms, experiential or social phenomena related terms, filters for qualitative research, and search limits) | 6 |
| 7. Study screening methods | Describe the process of study screening and sifting (e.g. title, abstract and full text review, number of independent reviewers who screened studies) | 6 |
| 8. Study characteristics | Present the characteristics of the included studies (e.g. year of publication, country, population, number of participants, data collection, methodology, analysis, research questions) | Supplement 2 |
| 9. Study selection results | Identify the number of studies screened and provide reasons for study exclusion (e,g, for comprehensive searching, provide numbers of studies screened and reasons for exclusion indicated in a figure/flowchart; for iterative searching describe reasons for study exclusion and inclusion based on modifications to the research question and/or contribution to theory development) | Figure 1 |
| 10. Rationale for appraisal | Describe the rationale and approach used to appraise the included studies or selected findings (e.g. assessment of conduct (validity and robustness), assessment of reporting (transparency), assessment of content and utility of the findings) | 6-7 |
| 11. Appraisal items | State the tools, frameworks and criteria used to appraise the studies or selected findings (e.g. Existing tools: CASP, QARI, COREQ, Mays and Pope [25]; reviewer developed tools; describe the domains assessed: research team, study design, data analysis and interpretations, reporting) | 6-7 |
| 12. Appraisal process | Indicate whether the appraisal was conducted independently by more than one reviewer and if consensus was required | 6-7 |
| 13. Appraisal results | Present results of the quality assessment and indicate which articles, if any, were weighted/excluded based on the assessment and give the rationale | Supplement 3 |
| 14. Data extraction | Indicate which sections of the primary studies were analysed and how were the data extracted from the primary studies? (e.g. all text under the headings “results /conclusions” were extracted electronically and entered into a computer software) | 7 |
| 15. Software | State the computer software used, if any | NA |
| 16. Number of reviewers | Identify who was involved in coding and analysis | 7-8 |
| 17. Coding | Describe the process for coding of data (e.g. line by line coding to search for concepts) | 7-8 |
| 18. Study comparison | Describe how were comparisons made within and across studies (e.g. subsequent studies were coded into pre-existing concepts, and new concepts were created when deemed necessary) | 7-8 |
| 19. Derivation of themes | Explain whether the process of deriving the themes or constructs was inductive or deductive | 8 |
| 20. Quotations | Provide quotations from the primary studies to illustrate themes/constructs, and identify whether the quotations were participant quotations of the author’s interpretation | 10-19  Table 1 |
| 21. Synthesis output | Present rich, compelling and useful results that go beyond a summary of the primary studies (e.g. new interpretation, models of evidence, conceptual models, analytical framework, development of a new theory or construct) | 10-19 |

# Supplement 2. *Characteristics of the primary studies*

| Study | Clients’ presenting issues / client population | N of therapists | Therapists’ age | Therapists’ gender | Country/Ethnicity | Therapists’ experience – general | Therapists’ experience – ROM | Treatment approach | Treatment setting | Data collection method | Data analysis method (N of analysts) | ROM system/ measure |
| --- | --- | --- | --- | --- | --- | --- | --- | --- | --- | --- | --- | --- |
| Antunes et al. (2020) |  | 4 |  |  | Portugal | Experienced | 2 to 4 years | EFT, CBT, group therapy | Private practice, psycho-oncology | Interview | QA | PQ |
| Bowens & Cooper (2012) | Adults | 10 | 18-65 | 2 M/8 F | UK | 6 experienced (M=8.8 years), 4 trainees | 3 had previous experience with TPF | Integrative, humanistic, PCA |  | Interview | TA | TPF |
| Brooks Holliday et al. (2020) | Trauma, substance use disorder, and other mental health issues / Adults | 60 (43 clinicians, 12 administrators, 5 support staff members) |  |  | USA |  | 34 extensive, 21 moderate, 4 no experience |  | U.S. Veterans Health Administration outpatient mental health care | Interview | TA | PHQ-9, PCL-5, GAD-7, BAM |
| Brooks Holliday et al. (2021) | Trauma, substance use disorder, and other mental health issues / Adults | 26 (19 psychologists, 3 psychiatrists, 1 social worker, 1 psychiatric nurse, 1 recreational therapist, 1 case manager) |  |  | USA |  | Each used MBC with at least one client |  | U.S. Veterans Health Administration outpatient mental health care | Interview | QA | PCL-5, PHQ-9, GAD-7, BAM |
| Callaly & Hallebone (2001) |  | 12 (3 psychiatrists, 3 team co-ordinators, 3 case managers and 3 service managers) |  |  | Australia |  |  |  |  | Interview | GT | BASIS-32 |
| Callaly et al. (2006) | Children, adolescents, and adults | 83 (64 psychiatric nurses, 12 allied health staff, 7 medical staff) |  |  | Australia |  |  |  | Mental health service | Group discussion, interview | GT | HoNOS, HoNOSCA, LSP, CGAS, FIHS, BASIS-32 |
| Coombs et al. (2011) |  | 6 individuals responsible for implemention |  |  |  |  |  |  |  | Interview |  | HoNOS, HoNOSCA, HoNOS65+, LSP-16, RUG-ADL, CGAS, MHI, BASIC-32, K-10+, SDQ |
| Dayton (2011) | Individual, group, and student couples / Adults | 22 | 30-60 | 14M/8F | European American | Some had less than 5 years of post-licensure experience while others were in the final 5 years of their career | Several years of using OQ-45 | Gestalt, REBT, DBT, ACT, CCT/PCA, psychodynamic, integrative, PCA, existential | University Counseling Center | Interview | PA | OQ-45 |
| De Beurs et al. (2011) | Mood, anxiety, and somatoform disorders | 20 |  |  | Netherlands |  | ≥4 years | CBT, medication, or combination | Mental health clinics, University Medical Center | Survey | QA | MINI-Plus, DAPP-SF, BDI-R, CGI, GAF |
| De Wilde Brand et al. (2022) | borderline personality disorder (BPD) / Adults | 10 (4 therapists with a post-master degree in Healthcare psychology, 3 psychotherapists, 2 psychologists, 1 psychiatrist ) |  | 2M/8F | Netherlands |  |  | Mentalization Based Treatment (MBT) | Dutch institute specialized in psychotherapy for personality disorders | Interview | TA | BSI, SIPP-sf), PDS, BPDSI-IV |
| Delgadillo et al. (2017) | Depression, anxiety | 15 |  |  | White British |  | 6-hour training | 16 CBT, 2 IPT | IAPT stepped care service | Interview | TA | PHQ-9, GAD-7 |
| Dias et al. (2016) |  | 14 | 25-43 (M=34.3) | 4M/10F | Portugal | 4 had <5 years, 3 had 5 to 10 years, and 7 had >10 years | 13 used the ROM app |  | Private Psychology Clinics, Private Therapeutic Centers, Private Hospitals, Psychology Service of a Private College | Interview | QA |  |
| Errázuriz & Zilcha-Mano (2018) | Adults of low and middle SES, mostly mood disorders / Adults | 28 psychologists | M=37.76y (SD=7.79) | 9M/19F | Chile | M = 8.38 (SD = 5.33) years | 31.87% had never used ROM prior to the study, 38.26% used them occasionally, and 29.87% used them on a regular basis | Orientation rated on a scale from 1 to 5: systemic M = 3.75 (SD = 1.16), cognitive M = 3.39 (SD = 1.41), psychodynamic M = 3.00 (SD = 1.60), behavioral M = 2.77(SD =1.63), and humanistic/existential M = 2.00 (SD =1.65) | Outpatient mental health center | Interview | CA | OQ 30.2, WAI, SCS |
| Esmiol-Wilson et al. (2017) |  | 26 | 21-48 years, (M= 29.63) | 2M/24F | USA, 19 white, 7 other | Trainees |  | marriage and family therapy | Training clinic | Interview | GT | ORS, SRS |
| Garland et al. (2003) | Children, adolescent, and adults | 50 (12 counselors, 16 social workers, 20 psychologists, 2 other) | M=39.7 (9.5) | 10M/40F | Caucasian 76, Hispanic 2, African American 6, Asian/Pacific Islander 10, Other 6 | Years experience 12.8 (8.4) |  |  | Outpatient treatment, day treatment, case management, residential treatment | Interview | QA | CBCL, YSR, CAFAS, CSQ |
| Gleacher et al. (2016) | Children, adolescent, and adults | 18 | 26-30 | 4M/14F | 10 Caucasian, 3 African-American, 5 Multi-Racial, and 2 Hispanic | 12 more experienced (licensed or >10 cases), 6 less experienced (<1 year) |  |  | Clinics at urban and rural area | Interview | CA | CFS |
| Hall et al. (2014) | Children and adolescents | 10 (5 clinical psychologists, 2 mental health nurses, 1 nurse prescriber, 1 consultant psychiatrist, and 1 trainee Psychiatrist) |  | 2M/8F | UK | 1–11 years (M = 7.0, SD = 3.5) |  |  | CAMHS | Interview | TA | SDQ |
| Hovland & Moltu (2019) |  | 18 | 25-60 | 6M/12F | Norway |  | 9 had 1 year experience, 9 had 3-6 month experience |  | Public outpatient and inpatient clinics | Interview | QA | NF |
| Hovland et al. (2023) | Adults | 18 (Individual therapists, milieu therapists) | 25-60 | 6M/12F | Norway |  |  |  | Public outpatient and inpatient clinics | Interview | SDI | NF |
| Ionita et al. (2016) | Children adolescents, adults, elderly, couples, families, groups | 25 (15 psychologists, 4 social workers, 2 counselors, 2 mental health workers, 2 marriage and family therapists) | 20-70 | 19M/6F | 21 lived in Canada or USA, 4 lived in Australia, New Zealand, UK, and India | From less than 2 years to 30+ years |  | Eclectic/integrative, humanistic, CBT, IPT/relational, systemic, experiential, narrative, existential, behavioral, dynamic |  | Interview | QA | ORS, SRS, OQ, Basis-32 |
| James et al. (2015) | Children, adolescents | 12 |  |  | UK |  |  |  | CAMHS | Interview | TA | CORC |
| Koementas-de Vos et al. (2022) | Mood and anxiety disorders / Adults | 9 | 27–43 (M = 35 years) | 1M/8F | 7 Dutch, 1 Caribbean, 1 Turkish Dutch | 8.0 years (SD = 5.0) | 6 to 16 sessions, none had used ROM in group therapy prior to this study | Group therapy (IPT and CBT) |  | Interview | TA | FIGT |
| Langley et al. (2010) | Middle school youth (grades 6–8) | 27 (10 clinical social workers, 4 marriage family therapists, 6 school psychologists, 2 clinical psychologists, 2 psychologists, 2 counselors, 1 school nurse) |  |  | USA |  | n=18 had 6 to 24 months experience,  n=9 did not implement ROM | CBT |  | Interview | QA | CBITS |
| Lavik et al. (2020) | Child and adolescent mental health setting | 34 (20 clinical psychologists, 6 psychiatrists, 5 clinical social workers, 3 clinical pedagogues) |  |  | Norway |  |  |  | Outpatient clinics | Interview | TA | NF, NFPC, NFA |
| Lucock et al. (2015) | More severe, complex, and enduring mental health problems, including personality difficulties and interpersonal problems / Adults | 42 |  |  | UK | 26 permanent and qualified therapists, 8 trainee clinical psychologists, and 8 other therapists |  | CBT, CAT, psychodynamic, integrative | NHS | Review meetings, survey | TA | CORE-10, ASC, HASQ, FRQ, FSQ, PEQ |
| MacMurray (2019) |  | 6 |  | 5M/1F | USA | Training directors, graduate program directors, clinic directors |  |  |  | Interview | NA | PCOMS, FIT, ACORN, OQ-45, CCAPS |
| Martin et al. (2011) | Child’s secondary mental health care | 50 |  | 13M/37F | UK | A few months to 22 years (Mdn = 7 years) |  |  | CAMHS | Interview | TA | DAWBA |
| Meehan et al. (2006) | Children, adolescent, and adults | 324 (in 34 focus groups; 169 nurses, 85 allied health professionals, 21 psychiatrist/MD) |  |  | Australia |  | 8 months | Adult and youth inpatients, adult and youth community, forensic/secure, rehabilitation |  | Interview | CA | OIS |
| Moltu et al. (2018) | Adults | 37 (6 clinical specialist psychologist, 6 resident psychologist, 5 psychiatrist, 1resident doctor 1, 15 specialized psychiatric nurse, 4 specialized psychiatric social worker) | 20–60 | 12M/25F | Norway | 3 had >5 years, 19 had 6–15 years, 15 had 16–30 years |  |  | Public hospital trust | Focus group | TA |  |
| Norman et al. (2014) | Children and adolescents | 50 (psychiatrists, psychologists, social workers, family therapists, psychotherapists, art therapists, occupational therapists, teachers, nurses, primary mental health workers) |  | 14M/36F | UK | A few months to 20 years |  |  | CAMHS | Interview | GT, CA | PHQ-9, GAD-7, SDQ, RCADS, ESQ |
| Savic & Fomiatti (2016) | Alcohol and drug abuse | 23 |  |  | Australia |  | 3 months |  | Short-term outpatient counseling (around six sessions) | Focus group | ICCM | ATOP |
| Sharples et al. (2017) | Children and adolescents | 9 |  |  | UK |  |  |  | CAMHS | Interview | TA | NR |
| Sichel & Connors (2022) |  | 80 | 21–61 |  | USA | From <1 year to 20+ years | 1 year |  | Community-based mental health agencies | Survey | TA | CFF |
| Southwick (2011) |  | 11 (psychologists) |  |  | USA |  |  | CBT, eclectic, psychodynamic, experiential | University-based counseling center | Interview | TA | CST |
| Stefancic et al. (2022) | Patients experiencing first-episode psychosis / Adolescents and young adults (aged 16-30 years) | 11 (5 psychiatric care providers, 3 team leaders, 3 therapists) |  |  | USA |  |  |  | Clinics delivering coordinated specialty care | Interview | MA | FREEDoM |
| Sundet (2012) | Conduct disorder, ADHD, OCD, and developmental or emotional problems / Children, adolescents, and adults | 4 | 47-63 | 2M/2F | Norway | 3 experienced clinicians (20+ years), 1 trainee |  |  | Combined day treatment and outpatient unit | Interview | CQR | SRS, ORS |
| Sundram et al. (2017) | Adolescents with mild-to-moderate depression | 50 (in 7 focus groups; general practitioners, school guidance counselors, clinical psychologists, youth workers, and nurses) |  |  | New Zealand |  |  |  | Youth health center, Primary care service, School guidance service | Focus groups | TA | SPARX |
| Tarp et al. (2022) | Anxiety disorders (group therapy) OCD (individual therapy) / Adults | 3 (psychiatric nurse, psychotherapist and social worker, psychologist specialized in psychotherapy) | 37-52 (M = 45) | 3F | Denmark | 1 to 22 years (M = 11) | 3m/5month | CBT | Mental health services | Interview | TA |  |
| Teruya et al. (2006) | Substance abuse treatment / Adults | 230 (159 counselors or substance abuse specialists, 35 administrators, 24 administrative assistants, 7 data processors, 5 others) |  | 83M/147F | USA | <1 to 30 years (M = 7) |  |  | Residential and outpatient | Focus groups | CA | ASI, ASAM PPC II |
| Trauer et al. (2009) |  | 61 (28 nurses, 12 doctors, 9 social workers, 7 psychologists, and 5 occupational therapists) |  |  | Australia | 1 to 42 years (M = 12.7, SD = 10.8) | 42 were trained in using ROM, 19 not |  | Mental health service | Field notes from team meetings |  | HoNOS, LSP-16, BASIS-32 |
| Unsworth et al. (2017) |  | 9 |  |  | UK | 5 experienced (M = 8 years), 4 trainees | 6+ months |  | NHS | Focus group | QA | CORE-Net |
| Van Wert et al. (2021) | Children, adolescents, and adults | 138 |  |  | USA | 4 had <1 year, 28 had 1 to 5 years, 28 had 5 to 10 years, 78 had 10+ years |  |  | Outpatient, intensive outpatient, psychiatric rehabilitation, case management, mobile treatment, and school-based services | Survey | TA |  |
| Whitcomb et al. (2018) | Relationship issues, anxiety, depression, self-esteem, adjustment, impulse control, stress, substance abuse, social skills, eating disorders, self-mutilation, and pornography / Adults | 16 |  |  | USA |  |  | CBT, humanistic, psychodynamic, systems, integrative | University counselling center | Written responses after every session, interview | CA | GQ, OQ-45 |
| Winkeljohn Black et al. (2017) | Depression and anxiety symptoms / Adults | 1 |  | F | White | Trainee |  | Psychodynamic–interpersonal |  | Interview |  | AiA, WAI, PCOMS |
| Wolpert et al. (2016) | Children | 4 |  |  | UK |  |  |  | Child mental health services | Interview | CA |  |
| Woodland (2015) | Generalized anxiety / Adults | 11 | M = 41.6 (SD = 11.1) | 8M/3F | USA (10 White/Caucasian, 1 Asian) | M = 12.3 years (SD = 11.9) |  | Humanistic/existential, interpersonal, Gestalt, psychodynamic, integrative, Quantum | University counselling center, process-oriented groups | Written responses, interview | CA, GT | GQ, OQ-45 |
| Zhou et al. (2020) |  | 247 (54.1% clinical psychologists, 21.8% school psychologists, and 15.2% counselling psychologists) |  | 140M/259F | 90% European-Canadian/White |  | Some |  | 43% worked in private practice | Survey | CQR | TFB |

*Note:* empty cell = not reported. **ACORN** = A Collaborative Outcomes Resource Network, **ACT** = Acceptance and Commitment Therapy, **AiA** = Alliance in Action, **ASAM PPC II** = American Society of Addiction Medicine Patient Placement Criteria form, **ASC** = The Assessment for Signal Cases, **ASI** = Addiction Severity Index Lite, **ATOP** = Australian Treatment Outcomes Profile, **BAM** = Brief Addiction Monitor, **BASIS-32** = Behaviour and Symptom Identification Scale, **BDI-R** = the Brief Symptom Inventory, **BSI** = Brief Symptom Inventory, **BPDSI-IV** = Borderline Personality Disorder Severity Index-IV, **CA** = Content analysis, **CAFAS** = Child and Adolescent Functional Assessment Scale, **CAMHS** = Child and Adolescent Mental Health Services, **CAT** = Cognitive Analytic Therapy, **CBCL** = Child Behavior Checklist, **CBITS** = Cognitive Behavioral Intervention for Trauma in Schools, **CBT** = cognitive-behavioral therapy, **CCAPS** = Counseling Center Assessment of Psychological Symptoms, **CCT** = Client-Centered Therapy, **CFF** = Client Feedback Form, **CFS** = Contextualized Feedback System, **CGAS** = the Children’s Global Assessment Scale, **CGI** = Clinical Global Impression, **CORC** = Outcomes Research Consortium, **CORE-10** = Clinical Outcomes in Routine Evaluation, **CORE-Net** = Clinical Outcomes in Routine Evaluation with computer software, **CQR** = Consensual Qualitative Research, **CSQ** = Client Satisfaction Questionnaire, **CST** = Clinical Support Tools, **DAPP-SF** = Dimensional Assessment of Personality Pathology-Short Form, **DAWBA** = Development and Well-Being Assessment, **DBT** = Dialectical Behavior Therapy, **EFT** = emotion-focused therapy, **ESQ** = Experience of Service Questionnaire, **FIGT** = Feedback-informed group treatment, **FIHS** = Factors Influencing Health Status, **FIT** = Feedback-Informed Treatment, **FREEDoM** = First Episode Digital Monitoring (an app-based mHealth intervention), **FRQ** = Feedback Response Questionnaire, **FSQ** = Feedback System Questionnaire, **GAD-7** = Generalized Anxiety Disorder-7, **GAF** = Global Assessment of Functioning, **GT** = Grounded theory, **HASQ** = Helpfulness Alliance and Stage Measure, **HoNOS** = The Health of the Nation Outcome Scales, **HoNOS65+** = Health of the Nation Outcome Scales, **HoNOSCA** = Scales for Children and Adolescents, **IAPT** = Improving Access to Psychological Therapies, **ICCM** = inductive constant comparison method, **IPT** = Interpersonal Therapy, **K-10+** = Kessler-10 Plus, **LSP** = Life Skills Profile, **MA** = matrix analysis, **MCT** = Metacognitive Therapy, **MHI** = Scale Mental Health Inventory, **Mini Plus** = International Neuropsychiatric Interview-Plus, **NA** = Narrative analysis, **NF** = Norse Feedback, **NFA** = Norse Feedback Adolescent, **NFPC** = Norse Feedback Primary Care, **NHS** = National Health Service, **OIS** = Outcomes Information System, **OQ** = Outcome Questionnaire, **ORS** = Outcome Rating Scale, **PA** = Phenomenological analysis, **PCA** = person-centered approach, **PCL-5** = PTSD Checklist for DSM–5, **PCOMS** = Partners for Change Outcome Management System, **PDS** = Post-traumatic Diagnostic Scale, **PEQ** = Patient Experience Questionnaire, **PHQ-9** = Patient Health Questionnaire-9, **PQ** = Personal questionnaire, **QA** = Qualitative analysis (not specified), **RCADS** = Revised Children’s Anxiety and Depression Scale, **REBT** = Rational Emotive Behavior Therapy, **RUG-ADL** = Resource Utilisation Groups – Activities of Daily Living, **SCS** = Self-Concealment Scale, **SDI** = stepwise-deductive-inductive method, **SDQ** = Strengths and Difficulties Questionnaire, **SIPP-sf** = Severity Indices of Personality Problems-short form, **SPARX** = Smart, Positive, Active, Realistic, X-factor thoughts, **SRS** = Session Rating Scale, **TA** = Thematic analysis, **TFB** = Test Feedback, **TPF** = The Therapy Personalisation Form, **WAI** = Working Alliance Inventory, **YSR** = Youth Self-Report.

# Supplement 3. *Quality assessment of the primary studies*

| Reference | Criterion | | | | | | | Score |
| --- | --- | --- | --- | --- | --- | --- | --- | --- |
|  | 1. An explicit theoretical framework and/or literature review | 2. Aims and objectives clearly stated | 3. A clear description of context | 4. A clear description of the sample and how it was recruited | 5. A clear description of methods used to collect and analyze data | 6. Attempts made to establish the reliability or validity of data analysis | 7. Inclusion of sufficient original data to mediate between evidence and interpretation |  |
| Antunes et al. (2020) | x | x | x |  | x | x | x | 6 |
| Bowens & Cooper (2012) | x | x | x | x | x | x | x | 7 |
| Brooks Holliday et al. (2020) | x | x | x |  | x | x |  | 5 |
| Brooks Holliday et al. (2021). | x | x | x |  | x | x | x | 6 |
| Callaly & Hallebone (2001) | x | x |  |  | x |  |  | 3 |
| Callaly et al. (2006) | x | x |  |  | x |  | x | 4 |
| Coombs et al. (2011) | x | x | x |  |  |  |  | 3 |
| Dayton (2011) | x | x | x | x | x | x | x | 7 |
| De Beurs et al. (2011) | x | x | x |  |  | x |  | 4 |
| De Wilde Brand et al. (2022) | x | x | x | x | x |  | x | 6 |
| Delgadillo et al. (2017) | x | x | x |  | x | x | x | 6 |
| Dias et al. (2016) | x | x | x |  | x | x | x | 6 |
| Errázuriz & Zilcha-Mano (2018) | x | x | x | x | x | x |  | 6 |
| Esmiol-Wilson et al. (2017) | x | x | x | x | x | x | x | 7 |
| Garland et al. (2003) | x | x | x | x | x | x | x | 7 |
| Gleacher et al. (2016) | x | x | x | x | x | x | x | 7 |
| Hall et al. (2014) | x | x | x |  | x | x | x | 6 |
| Hovland & Moltu (2019) | x | x | x |  | x | x | x | 6 |
| Hovland et al. (2023) | x | x | x |  | x | x | x | 6 |
| Ionita et al. (2016) | x | x | x | x | x | x | x | 7 |
| James et al. (2015) | x | x | x |  | x | x | x | 6 |
| Koementas-de Vos et al. (2022) | x | x | x | x | x | x | x | 7 |
| Langley et al. (2010) | x | x | x |  | x | x | x | 6 |
| Lavik et al. (2020) | x | x |  |  | x | x | x | 5 |
| Lucock et al. (2015) | x | x | x |  | x | x |  | 5 |
| MacMurray (2019) | x | x | x |  | x | x | x | 6 |
| Martin et al. (2011) | x | x | x |  | x | x | x | 6 |
| Meehan et al. (2006) | x | x |  |  | x | x | x | 5 |
| Moltu et al. (2018) | x | x |  |  | x | x | x | 4 |
| Norman et al. (2014) | x | x |  |  | x | x | x | 5 |
| Savic & Fomiatti (2016) | x | x | x |  |  | x | x | 5 |
| Sharples et al. (2017) | x | x | x |  | x | x | x | 6 |
| Sichel & Connors (2022) | x | x | x |  | x | x | x | 6 |
| Southwick (2011) | x | x | x |  | x | x | x | 6 |
| Stefancic et al. (2022) | x | x | x |  | x | x | x | 6 |
| Sundet (2012) | x | x | x |  | x | x | x | 6 |
| Sundram et al. (2017) | x | x | x |  | x | x | x | 6 |
| Tarp et al. (2022) | x | x | x | x | x | x | x | 7 |
| Teruya et al. (2006) | x | x | x | x | x | x |  | 6 |
| Trauer et al. (2009) |  | x | x |  |  |  |  | 2 |
| Unsworth et al. (2017) | x | x | x |  | x | x | x | 6 |
| Van Wert et al. (2021) | x | x |  |  | x |  |  | 3 |
| Whitcomb et al. (2018) | x | x | x |  | x | x | x | 6 |
| Winkeljohn Black et al. (2017) | x | x |  |  |  | x | x | 3 |
| Wolpert et al. (2016) | x | x | x |  | x |  | x | 5 |
| Woodland (2015) | x | x | x |  | x | x | x | 6 |
| Zhou et al. (2020) | x | x | x |  | x | x |  | 5 |
| Marginal % | 98% | 100% | 83% | 23% | 89% | 85% | 79% |  |

# Supplement 4. *Occurrence of meta-categories per study*

|  | 1a | 1b | 2a | 2b | 2c | 2d | 3a | 3b | 3c | 4a | 4b | 4c | 4d | 5a | 5b | 5c | 5d | 6a | 6b | 6c | 6d | Intensity | % |
| --- | --- | --- | --- | --- | --- | --- | --- | --- | --- | --- | --- | --- | --- | --- | --- | --- | --- | --- | --- | --- | --- | --- | --- |
| Antunes et al. (2020) |  |  | x |  |  |  |  |  |  |  |  | x |  |  |  |  |  | x |  | x |  | 4 | 19% |
| Bowens & Cooper (2012) | x |  | x | x | x | x | x | x |  | x | x | x |  |  |  |  |  | x |  |  |  | 11 | 52% |
| Brooks Holliday et al. (2020) | x | x | x | x |  | x | x |  |  |  |  | x | x |  | x |  | x |  | x | x | x | 13 | 62% |
| Brooks Holliday et al. (2021) | x | x | x | x |  | x | x |  |  |  |  |  |  |  |  |  | x | x |  |  |  | 8 | 38% |
| Callaly & Hallebone (2001) | x |  | x |  |  | x | x |  | x |  |  | x |  |  |  |  |  |  |  |  |  | 6 | 29% |
| Callaly et al. (2006) | x | x | x |  |  |  | x | x |  |  |  | x | x |  |  |  |  |  |  |  |  | 7 | 33% |
| Coombs et al. (2011) | x |  |  | x |  |  |  |  |  | x | x |  |  |  |  |  | x |  |  |  |  | 5 | 24% |
| Dayton (2011) |  |  | x | x |  | x |  |  |  | x |  |  |  |  | x |  |  |  |  |  |  | 5 | 24% |
| De Beurs et al. (2011) | x | x |  | x |  | x | x | x |  |  | x | x | x |  | x |  | x |  |  |  |  | 11 | 52% |
| De Wilde Brand et al. (2022) | x | x | x |  |  |  |  |  |  |  |  |  |  |  |  |  |  |  |  |  | x | 4 | 19% |
| Delgadillo et al. (2017) |  | x |  | x |  |  | x |  |  |  |  | x | x | x |  |  | x |  |  |  |  | 7 | 33% |
| Dias et al. (2016) | x |  |  | x |  |  | x |  |  |  |  |  |  |  |  |  |  |  |  |  | x | 4 | 19% |
| Errázuriz & Zilcha-Mano (2018) | x |  |  |  |  |  |  |  | x | x |  |  |  |  |  |  |  |  |  |  |  | 3 | 14% |
| Esmiol-Wilson et al. (2017) |  | x |  | x |  |  |  | x | x | x | x | x |  |  |  |  |  |  |  |  |  | 7 | 33% |
| Garland et al. (2003) | x | x |  |  |  |  | x |  |  |  |  | x |  |  |  |  | x |  |  |  |  | 5 | 24% |
| Gleacher et al. (2016) |  |  |  |  |  |  |  |  |  |  |  |  |  |  |  |  | x |  |  |  |  | 1 | 5% |
| Hall et al. (2014) | x | x |  |  |  |  | x |  | x |  |  | x | x |  |  |  |  | x |  | x | x | 9 | 43% |
| Hovland & Moltu (2019) |  |  |  |  |  |  | x |  |  | x |  |  |  |  |  |  | x |  |  |  |  | 3 | 14% |
| Hovland et al. (2023) | x | x |  |  | x |  | x |  |  |  | x | x |  |  | x |  |  |  | x |  |  | 8 | 38% |
| Ionita et al. (2016) |  |  |  | x |  |  |  |  |  |  |  |  |  |  |  |  | x |  |  |  | x | 3 | 14% |
| James et al. (2015) |  | x |  | x | x |  |  |  |  | x |  | x | x |  | x |  |  |  |  |  |  | 7 | 33% |
| Koementas-de Vos et al. (2022) | x | x |  |  |  |  |  |  | x |  |  | x | x | x | x |  | x | x | x |  |  | 10 | 48% |
| Langley et al. (2010) |  |  |  |  |  |  |  |  |  |  |  | x |  |  |  |  |  |  |  |  |  | 1 | 5% |
| Lavik et al. (2020) | x |  |  |  | x |  |  | x |  | x | x | x |  |  |  |  |  |  |  |  | x | 7 | 33% |
| Lucock et al. (2015) |  |  | x |  |  |  | x |  |  |  |  |  |  |  |  |  | x |  | x |  |  | 4 | 19% |
| MacMurray (2019) |  |  | x |  |  |  |  |  |  |  |  |  |  |  |  |  |  |  |  |  |  | 1 | 5% |
| Martin et al. (2011) | x |  | x |  |  |  | x |  |  |  | x |  |  |  |  |  |  |  |  | x | x | 6 | 29% |
| Meehan et al. (2006) | x | x |  |  |  |  |  |  |  |  |  |  |  |  |  |  | x |  |  |  |  | 3 | 14% |
| Moltu et al. (2018) | x |  | x |  | x |  | x | x |  |  | x | x |  |  |  |  |  |  |  |  | x | 8 | 38% |
| Norman et al. (2014) | x | x | x | x | x | x | x |  |  |  |  | x |  |  |  |  |  |  |  |  | x | 9 | 43% |
| Savic & Fomiatti (2016) |  | x |  |  |  |  | x |  |  |  |  |  | x |  |  | x |  | x |  |  | x | 6 | 29% |
| Sharples et al. (2017) |  | x | x |  |  |  | x | x | x |  |  | x |  |  |  |  |  | x |  |  |  | 7 | 33% |
| Sichel & Connors (2022) | x | x |  | x |  |  |  |  |  |  |  | x |  |  |  |  | x |  |  |  |  | 5 | 24% |
| Southwick (2011) | x |  |  |  |  |  | x |  |  |  |  |  |  |  |  |  |  |  |  | x |  | 3 | 14% |
| Stefancic et al. (2022) | x |  |  |  |  |  |  |  |  |  |  | x |  |  |  |  |  |  |  |  |  | 2 | 10% |
| Sundet (2012) |  | x |  | x | x |  | x | x | x | x | x |  | x |  |  | x |  |  |  |  | x | 11 | 52% |
| Sundram et al. (2017) | x |  |  |  |  |  | x |  |  | x |  | x |  |  |  |  |  |  |  | x |  | 5 | 24% |
| Tarp et al. (2022) | x | x | x |  |  |  | x |  |  |  |  |  | x |  |  |  |  |  |  |  |  | 5 | 24% |
| Teruya et al. (2006) | x |  |  |  |  |  |  |  |  |  |  | x |  |  |  |  |  | x |  |  |  | 3 | 14% |
| Trauer et al. (2009) | x | x | x |  |  | x |  |  |  |  |  |  |  |  |  |  | x | x |  |  |  | 6 | 29% |
| Unsworth et al. (2017) | x | x |  | x |  | x | x |  |  | x |  |  | x | x | x |  |  | x |  | x |  | 11 | 52% |
| Van Wert et al. (2021) | x |  |  |  |  |  |  |  |  |  |  |  |  |  |  |  | x |  |  |  |  | 2 | 10% |
| Whitcomb et al. (2018) | x |  |  | x |  |  | x |  |  | x |  |  |  |  |  |  |  |  | x |  |  | 5 | 24% |
| Winkeljohn Black et al. (2017) |  |  |  |  |  |  | x |  | x | x | x |  |  |  |  |  |  |  |  |  |  | 4 | 19% |
| Wolpert et al. (2016) |  | x | x |  |  |  | x |  |  |  |  | x |  |  |  |  |  |  |  |  |  | 4 | 19% |
| Woodland (2015) |  |  |  |  |  |  |  |  |  |  |  |  |  |  |  |  |  |  |  | x |  | 1 | 5% |
| Zhou et al. (2020) | x | x | x |  |  |  | x |  |  |  |  | x | x |  |  |  |  |  |  |  | x | 7 | 33% |
| Frequency | 30 | 23 | 18 | 16 | 7 | 9 | 27 | 8 | 8 | 13 | 10 | 24 | 12 | 3 | 7 | 2 | 15 | 10 | 5 | 8 | 12 |  |  |
| % | 64% | 49% | 38% | 34% | 15% | 19% | 57% | 17% | 17% | 28% | 21% | 51% | 26% | 6% | 15% | 4% | 32% | 21% | 11% | 17% | 26% |  |  |

*Note:* **Cluster 1:** 1b. Assessing Clients’ Momentary Status, 2b. Assessing Clients’ Progress; **Cluster 2:** 2a. Treatment Planning, 2b. Adjusting the Treatment Process, 2c. Structure Reminder, 2d. Case Management; **Cluster 3:** 3a. Opening and Speeding up Discussion, 3b. Supporting Difficult Conversations; **Cluster 4:** 4a. Allowing Clients to Feel Heard, 4b. Enhancing Transparency in the Therapeutic Relationship, 4c. Facilitating Clients’ Involvement, 4d. Nonspecific Impacts on the Therapeutic Relationship; **Cluster 5:** 5a. Facilitating Insight in Clients, 5b. Keeping Clients Focused, 5c. Reinforcing Positive Change, 5d. Other Therapeutic Impacts; **Cluster 6:** 6a. Adapting Timing of Administration, 6b. Adapting Mode of Administration, 6c. Adapting How Feedback is Discussed with Clients, 6d. Focusing on Specific Aspects, 6e. Contextual Interpretation. Frequency = in how many primary studies the meta-category was present. Intensity = to how many meta-categories a study contributed.
